# Telemedicine in the pediatric surgery in Germany during the COVID-19 pandemic

**DOI:** 10.1007/s00383-020-04822-w

**Published:** 2021-01-02

**Authors:** G. Lakshin, S. Banek, D. Keese, U. Rolle, A. Schmedding

**Affiliations:** Department of Pediatric Surgery and Pediatric Urology, University Hospital, Goethe University Frankfurt, Theodor-Stern-Kai 7, 60590 Frankfurt am Main, Germany

**Keywords:** Telemedicine, Pediatric surgery, COVID-19, Expansion, Satisfaction

## Abstract

**Purpose:**

The COVID-19 pandemic has led to an unprecedented expansion of telemedicine services worldwide. This study aimed to explore the practice of telemedicine in Pediatric Surgery in Germany, the impact of the pandemic on its development and parents’ and surgeons’ experiences with telemedicine.

**Methods:**

The study is a cross-sectional analysis using three surveys between 6/2020 and 10/2020: (1) all Pediatric Surgery departments of Germany reported whether they provide telemedicine services. (2) Members of the German Society of Pediatric Surgery and (3) families who participated in an outpatient visit by telephone or video with the Department of Pediatric Surgery and Pediatric Urology of the University Hospital Frankfurt completed an anonymous survey on their experience with telemedicine.

**Results:**

21% of the Pediatric Surgery departments in Germany provided telemedicine, of which 57% started due to the pandemic. The lack of physical examination and face-to-face contact seem to be the major limitations to surgeons and parents. 48% of the parents answered that telemedicine is equal to or better than traditional appointments, while 33% thought that telemedicine is worse.

**Conclusions:**

This study shows that families and doctors alike have had positive experiences with telemedicine and most will continue to use this format after the pandemic.

## Introduction

Telemedicine is a versatile toolset for providing healthcare defined as “the use of electronic information and communications technologies to provide and support health care when distance separates the participants” [1]. The technologies range from standard telephone audio consultations [2] to virtual reality scenarios [3, 4] and even drones [5]. Though these technologies have been available for some time, telehealth has not been widely used in German healthcare for legal, technical, and organizational reasons [6].

The COVID-19 pandemic has restricted patient access to hospitals and clinics, which has prompted an unprecedented expansion in telemedicine services [7–9] worldwide [10, 11].

This study was designed to analyze the evolution of telemedicine in the field of Pediatric Surgery in Germany. In March 2020, hospitals were instructed to restrict consultations and treatments to urgent or emergency cases only in response to the rapid rise in COVID-19 cases. Telemedicine emerged as an effective means for pediatric surgeons to avoid interrupting treatment for their elective patients.

We aimed to investigate the prevalence of telemedicine throughout Germany and to survey pediatric surgeons and their patients regarding their experiences and attitudes toward telemedicine. The purpose of this study was to provide insight into the spread, effectiveness, and future of this emerging medical practice.

## Materials and methods

Our study defined telemedicine as consultations performed remotely using audio or video communication technology. This study included three parts: (1) telemedicine in Pediatric Surgery departments throughout Germany; (2) pediatric surgeons’ experiences and perspectives; and (3) patients’ experiences and perspectives (3).

### Telemedicine in pediatric surgery departments throughout Germany

All of the Pediatric Surgery departments in Germany are listed on the homepage of the German Society of Pediatric Surgery (DGKCH). The 89 department heads were contacted by email and asked whether they provide telemedicine services (over the phone or in a video conference) and if so, whether these services were being offered in direct response to the COVID-19 pandemic.

### Pediatric surgeons’ experiences and perspectives

An anonymous survey was sent to the 812 members of the DGKCH. Completed surveys were accepted from January 8, 2020, to October 31, 2020. The online survey was distributed through the official forum of the DGKCH (as a link), included 34 questions on telemedicine practices, and required approximately 5 min to complete. The questions differed for those who provided and who did not provide telemedicine. Additional questions covered their experiences with tele- or videoconferencing with patients’ families, statistical information about the Pediatric Surgery facilities, their overall satisfaction and the likelihood to continue using telemedicine after the COVID-19 pandemic.

### Patients’ experiences and perspectives

All of the 120 families with telemedical appointments at the Department of Pediatric Surgery of the University Hospital of Frankfurt from 16.03.2020 to 30.06.2020 were identified using electronic medical scheduling records. A questionnaire was developed by the authors in both online and paper formats and sent to the families by email or post, respectively, after receiving verbal consent by telephone. The answers were provided anonymously. The families were asked 38 qualitative and quantitative (single-choice, multiple-choice, and open-ended) questions about their experience with telemedicine. These included questions about the practice of telemedicine in general and their tele- or videoconferencing visit specifically. Additional qualitative questions covered their experiences during the appointment, the protection of privacy and medical data, and a final overall rating of telemedicine.

### Data analysis

Descriptive statistical data are presented in total numbers as well as in relative percentages.

## Results

### Telemedicine in pediatric surgery departments throughout Germany

Of the 89 Pediatric Surgery departments listed by the German Society of Pediatric Surgery (DGKCH), 73% (65/89) responded to our request. Of these 65, 29% (19/65) provided telemedicine services, 20% (13/65) had video visits, 20% (13/65) had telephone visits and 11% (7/65) had both. 3% (2/65) planned on starting to provide telemedicine, while 71% (46/65) did not have telemedicine services. 85% (11/13) of the departments had launched a telemedicine service because of the COVID-19 pandemic specifically. University hospitals reported a higher utilization of telemedicine than the non-university hospitals (Table 1).

**Table 1:** Distribution of telemedical services in Pediatric Surgery in Germany

	Answers	Telemedicine	Video	Telephone	Started during pandemic
All hospitals *n* = 89	65 (73%)	19 (21%)	13 (15%)	13 (15%)	11 (12%)
University hospitals *n* = 33	26 (79%)	11 (33%)	9 (27%)	8 (24%)	7 (21%)

### Pediatric surgeons’ experiences and perspectives

10% (81/812) of the pediatric surgeons (members of the DGKCH) responded to the anonymous online questionnaire. Of this 81, 15% (12/81) pediatric surgeons had telephone-based consultations at their facilities and 12% (10/81) were active participants. 11% (9/81) of the surgeons had video appointments at their facilities and 6% (5/81) were active participants. 14% (11/81) had both audio and video, 11% (9/81) had personal experience with the practice. Most of the responding pediatric surgeons (59%, 54/81) reported that their facility did not provide telemedicine services. See Table 2 for additional details.

**Table 2:** Provision of telemedicine and professional characteristics of pediatric surgical study participants (*n* = 81) (*n.a.* no answer)

	No	Telephone visits	Video visits	Both	Start because of COVID	Plan to continue after the pandemic	Consider continuing after the pandemic
All	81	12 (15%)	9 (12%)	11 (14%)	18 (22%)	22 (27%)	9 (11%)
Position							
Head surgeon	28 (36%)	5 (18%)	3 (11%)	6 (21%)	13 (46%)	10 (36%)	4 (14%)
Consultants	21 (27%)	1 (5%)	1 (5%)	3 (14%)	3 (14%)	5 (24%)	1 (5%)
Specialist registrar	10 (13%)	2 (20%)	1 (10%)	1 (10%)	2 (20%)	3 (30%)	0
Interns	7 (9%)	2 (29%)	0	1 (14%)	2 (29%)	1 (14%)	2 (29%)
Pediatric surgeons in an ambulatory setting	12 (15%)	2 (17%)	4 (33%)	0	4 (33%)	2 (17%)	2 (17%)
No answers	3					1	0
Place of work							
University hospital	31 (40%)	5 (16%)	3 (10%)	9 (29%)	9 (29%)	13 (42%)	4 (13%)
Non-university hospitals	34 (43%)	5 (15)	2 (6%)	2 (6%)	5 (15%)	5 (15%)	3 (9%)
Ambulatory healthcare centers	2 (3%)	0	0	0	n.a	0	1 (50%)
Private practices	11 (14%)	2 (18%)	4 (36%)	0	4 (36%)	4 (36%)	2 (18%)
No answers	3						1

Of those who did not provide telemedicine, 37% (17/47) stated that the possibility of providing such services was discussed in their teams and 60% (28/47) said it was not. 60% (28/47) of surgeons could imagine themselves providing telemedicine while the remaining 40% (19/47) could not. 15% (7/47) stated that this possibility has been rejected by their institutions, while 67% (31/47) reported that it had not yet been rejected. In 9% (4/46) of the cases, telemedicine was planned for the future and in 85% (38/46) it was not. The rest did not give an answer.

30% (7/23) of the surgeons providing telephone consultations and 56% (10/18) of those with video consultations treated new patients or patients with new diagnoses. Of those who do not provide telemedicine yet, 45% (21/47) would consult new patients at least in some diagnoses, and 55% (26/47) would not consult unknown patients.

**Fig. 1:**
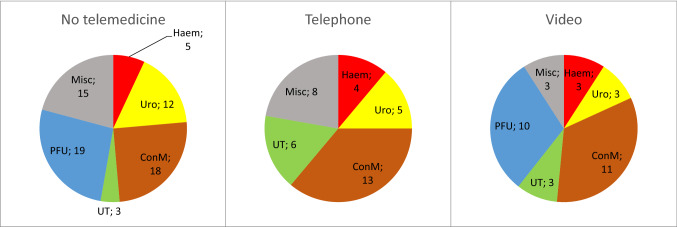
Most common telemedical diagnoses. From left to right: diagnoses by surgeons who do not practice telemedicine (left), diagnoses by those practicing telemedicine more often over the telephone (center) and in video visits (right). *Haem* hemangioma, *Uro* urology, *ConM* congenital malformations, *UT* micturition/defecation disorders, *PFU* post-traumatic follow-ups, *Misc* miscellaneous

Pediatric surgeons were asked to provide the three main diagnoses treated by telemedicine. Those, who were not practicing telemedicine were asked to propose three suitable diagnoses to be treated by telemedicine. We sorted the diagnoses into related groups: congenital malformations, post-traumatic follow-ups, micturition/defecation disorders, urology, hemangioma, and miscellaneous. These results are presented in Fig. 1.

24% (11/47) of those without telemedical experience in their department found it imaginable to indicate surgery remotely, 65% (30/47) found it unimaginable, while 13% (6/47) were not sure. 22% (5/23) of the surgeons had indicated surgery during telephone consultations and 39% (7/18) of the surgeons during video consultations.

Technical problems were reported as common by 11% (2/19), rare in 58% (11/19), and never in 32% (6/19) for telephone consultations. Technical problems for video consultations occurred commonly in 6% (1/16) and rarely in 94% (15/16). Of those whose facilities provide telephone visits, 82% (19/23) of the doctors thought positively about protection of private data during the visit, 9% (2/23) negatively, and 9% (2/23) were not sure. For video visits, the numbers were 84% (16/19) positive, 5% (1/19) negative, and 11% (2/19) unsure. 46% (22/47) of the pediatric surgeons who did not provide telemedicine were positive about the data protection during telemedical consultations, 15% (4/47) were negative, and 39% (18/47) were unsure.

91% (21/23) of the surgeons providing telephone visits think that patients are satisfied with the service, 89% (17/19) of those with video visits, and the rest could not tell (Fig. 4). The average of the overall rating of telemedicine on a scale from 1 (satisfied) to 6 (unsatisfied) was 2.22.

### Patients’ experiences and perspectives

**Fig. 2:**
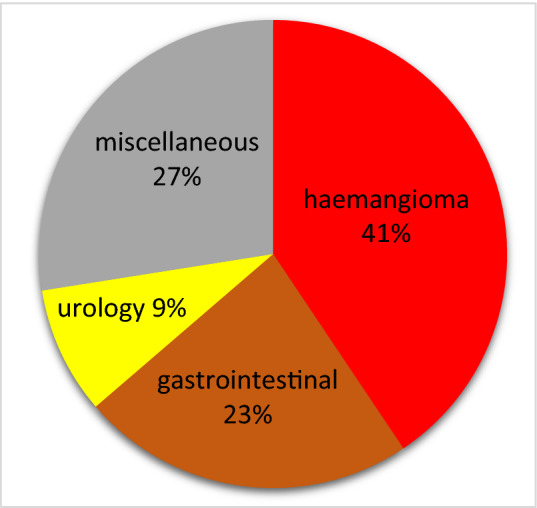
Most frequent diagnoses of telemedical patients in Frankfurt

86 (71.6%) of 120 total families responded to our anonymous survey in both online and paper forms. The statistics of the parents who filled out the questionnaire is given in Table 3. There were four major patient groups according to their diagnosis (Fig. 2): hemangioma (37/86), gastrointestinal disorders (21/86), urological diagnoses (8/86), miscellaneous (25/86).

**Table 3:** General data about the parents’ group

Who filled the survey	Mother	60	70%
	Father	23	27%
	Other family member	1	1%
	Unknown	2	2%
Highest educational qualification of either of the parents	Primary education	15	17%
	High-school education	15	17%
	University diploma	51	59%
	Unknown	5	6%
Age in years	< 30	16	19%
	30–44	65	76%
	> 44	5	6%

All but one patient’s family engaged with telemedicine by telephone. 96% (80/83) of the patients found the connection quality during their telephone consultations sufficient. 97% (76/78) experienced no technical problems during the call. 35% sent complimentary data via email (29/30) or using their smartphone (2/30). 100% (83/83) were confident that their privacy was protected.

35% (29/83) of the parents reported lacking visual contact with the doctor to be a disadvantage, while 61% (51/83) found it tolerable. 91% (74/81) were not bothered by not being able to see the doctor during the appointment. 96% (78/81) of the families trusted the physician. The medical content of the conversation was sufficiently explained in 95% (78/82) and the therapeutic measures in 93% (77/82) of cases. 88% (72/81) said the doctors could empathize with the patient while 11% (9/81) said they could not.

**Fig. 3:**
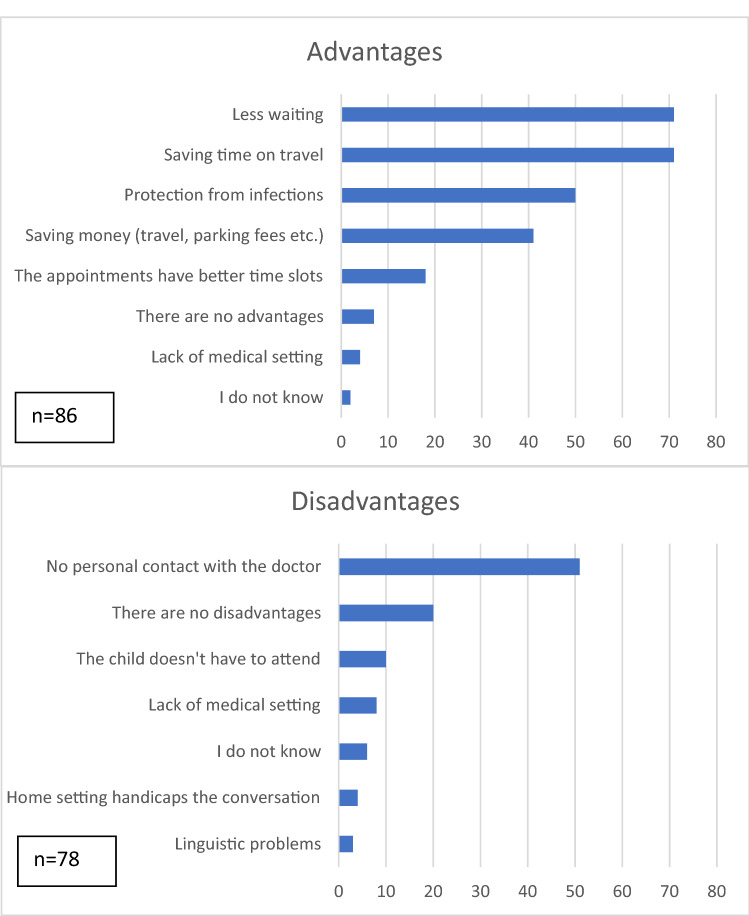
Disadvantages and advantages of telemedicine according to the families. The graphically-presented values represent raw numbers

The disadvantages and advantages of telemedicine are given in Fig. 3. When asked to compare a telemedical visit to a traditional, in-person one, 33% (28/84) found it inferior, 44% (37/84) found it to be equal, 4% (3/84) said it was superior while 19% (16/84) could not tell.

We also asked the families to describe the advantages and disadvantages of telemedicine in an open-ended question. Twelve advantages were provided. 50% (6/12) of the answers concerned saving time and resources. 25% (3/12) of the parents admired no necessity of the child’s participation and thus sparing exposure to stress or boredom. The other parents mentioned reliability, positive spontaneity, and empathy as features of the experience.

29 disadvantages were reported. 69% (20/29) of the parents mentioned the lack of a physical examination in their responses. 17% (2/29) questioned the quality of the doctor’s assumptions about the well-being of the patient while being influenced by the non-professional judgment of the parents. One parent found it hard for the child to participate in the conversation due to anxiety associated with speaking to someone they do not personally know. Two of the parents stated that some of the agreed-upon measures were forgotten by the doctors, e.g., sending a prescription or calling them back.

Families were asked to rate their satisfaction with telemedicine using a five-point Likert-type scale with one indicating “very satisfied” and five “very dissatisfied”. The average of the rating was 1.91.

## Discussion

Telemedicine has been utilized for decades [2]. Telemedicine plays a supportive role with conventional means of providing healthcare in suitable situations [12] and adjusts its scale and features to the task at hand [13, 14]. Telemedicine has been applied in the field of Pediatric Surgery [15] and pediatrics for a long time as well [16, 17] and was already on a trajectory of exponential global growth [18]. The COVID-19 pandemic has boosted the utilization of telemedicine greatly [19].

Telemedicine requires available healthcare, access to technology, legal infrastructure, and adequate financing [11, 20]. Furthermore, a combination of external stimuli [21–23] (such as the pandemic), the doctor’s willingness to provide it, and the patient's willingness to receive it are required.

Germany has highly available medical services at 4.49 medical doctors per 10.000 citizens [24] and a health insurance system that covers the vast majority of the population [25]. The first statutory regulation of telemedicine in Germany was published in 2015. Right before the outbreak of the COVID-19 pandemic, in November 2019, a major legal boost, the Digital Healthcare Act (Digitale-Versorgung-Gesetz) was published and became effective in December 2019. After the outbreak of the pandemic, the National Association of Statutory Health Insurance Physicians (Kassenärtzliche Bundesvereinigung) lifted some of the still-existing billing-related limitations for providing telemedicine services [26]. Concurrently, hospitals were requested to limit in-person visits to emergencies only.

The pandemic increased the number of telemedicine services in Pediatric Surgery by 50%, according to our survey.

The feedback provided by the members of the German Society of Pediatric Surgery was largely positive. They were open to telemedicine and confident about its capabilities, data protection issues, and foremost, the satisfaction of the patients. The views expressed by the medical professionals in this study are consistent with the literature [27, 28].

Our patients and their families were as satisfied with telemedicine services as the medical professionals. Given that the implementation of telemedicine in Germany is not standardized, our findings hint that, in general, it provokes a similar reaction from all of the participants. The strongest advantages of telemedicine were found to be saving time and resources, which are also the most mentioned aspects in the literature [29]. These aspects were so important to the families that they placed them above being protected from a potential infection during an in-person visit. This is remarkable given that the timing of the survey was during the ‘first wave’ of the COVID-19 pandemic.

Our data show that families and doctors share similar concerns about the disadvantages of telemedicine. The lack of physical examination and face-to-face contact seem to be the major limitations. The families fear that the doctor’s judgment of the situation, which relies heavily on the information provided by the parents, may be limited. The research on this topic appears to be consistent with the literature [30]. Videoconferencing seems more suitable for consulting with new patients as well as when indicating surgery compared to telephone consultations, while the majority of those surveyed indicated that they would not consult new patients or indicate surgery over the telephone but would indicate surgery in a video consultation.

The groups of most common diagnoses for teleconsultations were congenital malformations, urology, micturition/defecation disorders, hemangioma, post-traumatic follow-ups. Especially pediatric urological diagnoses have been proven to be suitable for telemedicine [7, 31–33], as well as post-traumatic follow-up [32]. For congenital malformations, telemedicine already plays a role in the antenatal evaluation [32, 34]. Further research on the possibilities of telemedicine in the postoperative care and long-term follow-ups of congenital malformations could be made.

Once again [32, 35, 36], our study indicates that telemedicine is applicable in the field of Pediatric Surgery. Patients, their families, and pediatric surgeons in Germany are increasingly tending to accept telemedicine as a legitimate tool.

### Limitations

The present study has some limitations. All of the patients included had telephone consultations and only one video patient was represented. The education level of the parents is higher than in the general population, which could render them more accepting of and positive toward this technology.

We did not acquire data on the number of telemedical consultations performed in the surveyed clinics, thus, our data is hard to compare with the literature in this regard.

## Conclusions

Germany has seen an increase in telemedicine services in the field of Pediatric Surgery during the COVID-19 pandemic. Both families and doctors place a great deal of trust in telemedicine. Despite its known limitations, many medical situations can be solved remotely. Despite all of the advantages and disadvantages of telemedicine, both families and doctors would continue using it after the COVID-19 pandemic. Telemedical services are a valuable addition to conventional outpatient visits.
